# Efficacy and safety of ashwagandha root extract on sexual health in healthy Men: a prospective, randomized, double-blind, placebo-controlled study

**DOI:** 10.3389/frph.2026.1774098

**Published:** 2026-02-12

**Authors:** Aman Khanna, Mallika Khanna, Parth Panchal

**Affiliations:** 1 Department of Internal Medicine, Aman Hospital and Research Center, Vadodara, Gujarat, India; 2 Department of Clinical Research, Aman Hospital and Research Center, Vadodara, Gujarat, India

**Keywords:** aphrodisiacs, randomized controlled trial, semen analysis, sexual function, sperm motility, withania somnifera

## Abstract

**Introduction:**

Ashwagandha (*Withania somnifera*) is widely recognized in Ayurvedic medicine as a potent Rasayana and aphrodisiac herb, with preclinical studies demonstrating androgen-modulating, anxiolytic, and antioxidant properties that may enhance male reproductive physiology. The present study aimed to rigorously evaluate the efficacy and safety of a standardized Ashwagandha Root Extract (ARE) in improving sexual function in healthy adult men.

**Methods:**

A prospective, randomized, double-blind, placebo-controlled, parallel-group clinical trial was conducted over 8 weeks in 76 healthy males aged 30–50 years. Participants were randomized (1:1) to receive either 300 mg ARE twice daily or a matched placebo. Sexual functioning was evaluated using validated instruments, including the Sexual Desire Inventory-2 (SDI-2), number of Satisfying Sexual Events (SSEs), and the International Index of Erectile Function (IIEF). Semen parameters were analyzed using WHO-standardized procedures, and quality of life was assessed with the Short Form-12 Health Survey. Both intention-to-treat and safety analyses were performed with a significance threshold of *α* = 0.05.

**Results:**

ARE supplementation resulted in statistically significant improvements across multiple domains of sexual function compared with placebo, including SDI-2 scores, SSEs, sexual desire, and overall IIEF outcomes (*p* ≤ 0.001). Semen analysis demonstrated a 36% increase in ejaculate volume, 38% improvement in total sperm count, and an 87% increase in total sperm motility after 8 weeks, with moderate to large effect sizes indicating clinically meaningful benefits. No adverse events or safety concerns were reported.

**Discussion:**

These findings suggest that standardized ARE may serve as an effective and well-tolerated natural intervention to support male sexual health.

**Clinical Trial Registration:**

https://ctri.nic.in/Clinicaltrials/pmaindet2.php?EncHid=NzY3ODE=&Enc=&userName=, identifier CTRI/2,022/11/047,501.

## 1 Introduction

The World Health Organization (WHO) defines sexual health as “a state of physical, emotional, mental, and social well-being in relation to sexuality, and not merely the absence of disease, dysfunction, or infirmity” (1). Optimal sexual functioning is integral to physical, psychosocial, and emotional well-being and is increasingly recognized as an important indicator of overall general health (2).

Sexual desire and performance are influenced by a complex interconnection of biological, psychological, and social factors. Among these, anxiety and psychological stress are major contributors to reduced libido and impaired sexual arousal (3). With the rapid pace of modernization and changing lifestyle patterns, the global prevalence of stress and anxiety has risen sharply, particularly among young adults (4). Parallel to this trend, an escalation in the incidence of male sexual dysfunction (MSD) has been reported. MSD typically encompasses low libido, erectile dysfunction (ED), Peyronie's disease, and disorders of ejaculation and orgasm. These conditions can negatively affect intimate relationships, contribute to unconsummated marriages, and lead to infertility, thereby imposing significant psychosocial burden (5).

Current therapeutic approaches for ED and related dysfunctions include pharmacological agents, surgical interventions, psychosexual counseling, and lifestyle modification strategies. However, conventional pharmacotherapy is associated with multiple systemic side effects (e.g., headache, flushing, hypotension, dyspepsia, nasal congestion, visual disturbances), which has increased the need for safer, holistic, and better-tolerated alternatives (6, 7).

Ashwagandha (*Withania somnifera*), a key medicinal plant of the Solanaceae family, is extensively used in Ayurveda and classified as a potent “Rasayana” with adaptogenic and aphrodisiac attributes (8). Evidence from clinical and preclinical research indicates that standardized *W. somnifera* root extract is safe, well-tolerated, and effective in improving oligospermia and related reproductive impairments (9, 10). Mechanistic studies suggest that Ashwagandha enhances seminal quality by optimizing essential metabolites such as amino acids, citrate, and lactate in seminal plasma. Additionally, its antioxidant, stress-reducing, and endocrine-modulating properties contribute to improved spermatogenesis and reproductive function (8). Mahdi et al. demonstrated that *W. somnifera* supplementation alleviates stress and improves semen parameters in males, while other investigations similarly report favorable effects on male sexual performance and reproductive health (11).

Considering the growing interest in botanical interventions for sexual wellness and the plausible mechanistic rationale for Ashwagandha, this prospective 8-week clinical trial was undertaken to evaluate the efficacy and safety of standardized Ashwagandha Root Extract (ARE) in improving sexual health among adult men.

## 2 Materials and methods

### 2.1 Study design and setting

This 8-week, prospective, double-blind, randomized, placebo-controlled, two-arm, parallel-group clinical trial was designed to evaluate the efficacy and safety of a standardized ARE in improving sexual health in adult men. This study was designed as a confirmatory randomized controlled trial for clinical sexual function outcomes, while hormonal and mechanistic endpoints were considered secondary and exploratory. The study was conducted in accordance with the principles of Good Clinical Practice (GCP) and the Declaration of Helsinki. The study protocol was reviewed and approved by the Institutional Ethics Committee (IEC) (Ref. #: AHRC/IEC/11/2022; dated 04 October 2022), and the trial was registered with the Clinical Trials Registry of India (CTRI/2022/11/047501; 22 November 2022; https://ctri.nic.in/Clinicaltrials/pmaindet2.php?EncHid=NzY3ODE=&Enc=&userName=). The Consolidated Standards of Reporting Trials (CONSORT) guidelines were followed in the design and reporting of the study. Written informed consent was obtained from all participants before any study-related procedures. The study was conducted between 06 December 2022 and 27 May 2023.

### 2.2 Eligibility criteria

A total of 76 healthy men aged 30–50 years, reporting to the study site for concerns related to sexual functioning, were screened for eligibility. Men who reported poor sexual satisfaction during the previous three months were assessed using the International Index of Erectile Function (IIEF) (12). Those who were in a stable, monogamous, heterosexual relationship were eligible for enrollment. Participants were otherwise healthy adult men without systemic, endocrine, neurological, or organic causes of erectile dysfunction, who reported mild-to-moderate sexual dysfunction as indicated by baseline IIEF scores between 11 and 16. Participants were required to abstain from using any medications or supplements intended to improve sexual performance during the study period.

All participants and their partners were required to be physically present for at least 50% of each calendar month and to be willing to engage in regular sexual intercourse. A medically approved form of contraception was mandated throughout the study. Men were also required to have a reliable and compliant partner who agreed to support adherence to study procedures. The study expected participants to attempt sexual intercourse at least four times within each two-week period. Only those who demonstrated adequate understanding and the ability to communicate openly about their sexual functioning with the study investigators, and who were willing to comply with the protocol and complete investigational product intake through Day 56 ± 3, were enrolled.

Men with any acute illness, clinically significant medical conditions, or any history that could compromise participant safety or study outcomes were excluded. Additional exclusion criteria included participation in other clinical studies involving dietary supplements; history of hypersensitivity reactions; known substance abuse or dependence; primary hypoactive sexual desire disorder; erectile dysfunction secondary to other primary sexual disorders; or a history of pelvic surgery-related sexual dysfunction. Men with spinal cord injury, radical prostatectomy, penile deformities, penile implants, malignancy, refractory psychiatric disorders, or significant neurological abnormalities were excluded. Alcohol addiction or persistent substance abuse were also excluded. Individuals intending to father a child during the study or those with pregnant partners were not eligible.

### 2.3 Randomization and blinding

A unique screening number was assigned to each participant after providing written informed consent. Once eligibility was confirmed, participants were randomized in a 1:1 ratio to receive either ARE 300 mg capsules or identical placebo. Randomization was performed using a computer-generated list (Rando version 1.2, Windows). Both study products were identical in size, shape, colour, and packaging to ensure complete blinding.

Randomization codes were generated by an independent statistician using the SNOSE (Sequentially Numbered, Opaque, Sealed Envelopes) method. Each envelope was sequentially numbered according to the randomization schedule, opaque to prevent visual inspection, and sealed to maintain allocation concealment. The envelopes were prepared by an independent statistician not involved in the conduct, assessment, or monitoring of the study. The investigator was instructed to open the respective SNOSE envelope only after assigning the participant's unique study number. This ensured that both participants and study personnel including investigators, site staff, and outcome assessors remained blinded to treatment allocation.

An independent and blinded investigator collected all outcome measures at each scheduled visit. Emergency unblinding was allowed only in the event of a serious adverse event (SAE) or medical emergency. In such cases, the principal investigator could request unblinding by accessing the participant-specific allocation code maintained in a secure, password-protected database and duplicate master SNOSE set. Every unblinding event required documentation and reporting to the IEC within 24 h.

### 2.4 Intervention

Participants in the Ashwagandha group received capsules containing standardized Ashwagandha Root Extract (KSM-66 Ashwagandha®, 300 mg), manufactured by Ixoreal Biomed Inc. (Los Angeles, California, USA). The capsules were administered orally twice daily, after breakfast and dinner, with a glass of water, for a duration of eight weeks. KSM-66 is a high-concentration, root-only extract produced using the green chemistry principles, an aqueous-based extraction process free from alcohol and chemical solvents. The extract contains >5% withanolides quantified by high-performance liquid chromatography (HPLC) and has a drug-to-extract ratio of 12:1. It is a light yellowish, neutral-tasting powder. The placebo group received identical capsules containing 300 mg starch. Both the active product and placebo were indistinguishable in color, shape, size, and overall appearance. All participants were instructed to maintain their usual diet and physical activity levels throughout the study.

### 2.5 Outcome measures

#### 2.5.1 Primary outcomes

The primary efficacy endpoints were the mean changes in Sexual Desire Inventory-2 (SDI-2) and Satisfying Sexual Events (SSEs) from baseline to Weeks 4 and 8. To minimize recall and social desirability bias, all questionnaires were self-administered in a private setting using validated instruments, and participants were assured of confidentiality.

##### 2.5.1.1 Sexual desire inventory-2 (SDI-2)

SDI-2 is a validated 14-item questionnaire that assesses dyadic and solitary sexual desire. Items were derived from theoretical models of sexual desire and DSM-III-R diagnostic criteria for Hypoactive Sexual Desire Disorder. Each item is scored on a Likert scale, with higher scores reflecting greater sexual desire (13). The questionnaire was self-administered at baseline and at each follow-up visit (Weeks 4 and 8) in a private setting to minimize reporting bias.

##### 2.5.1.2 Satisfying sexual events (SSEs)

SSEs were assessed based on the participant's diary and self-report. Participants recorded the total number of sexual intercourse attempts, successful vaginal penetration, successful intercourse duration, non-intercourse sexual interactions, number of orgasms, and the overall number of satisfying sexual activities during each assessment period (14).

Sexual desire was additionally scored using a 4-point Likert scale (0 = No desire; 1 = Mild; 2 = Moderate; 3 = Strong) based on the 24 h preceding each study visit. At baseline, sexual activity was recorded by recall for the preceding two weeks, while follow-up activity was recorded using a daily sexual activity diary, collected at Weeks 4 and 8.

#### 2.5.2 Secondary outcomes

Secondary efficacy endpoints included changes in International Index of Erectile Function (IIEF), seminal parameters, serum hormone levels, and quality of life (QoL) from baseline to Weeks 4 and 8.

##### 2.5.2.1 International Index of erectile function (IIEF)

IIEF is a validated, self-administered, 15-item questionnaire assessing four domains: Erectile Function (EF), Orgasmic Function (OF), Sexual Desire (SD), and Intercourse Satisfaction (IS). Each item is scored from 0 to 5, and domain scores are summed to yield a total IIEF score (12). Participants completed the IIEF during each visit in a private counseling room under study staff supervision.

##### 2.5.2.2 Quality of life (QoL)

QoL was assessed using the 12-item Short Form Health Survey (SF-12), which yields Physical Component Summary (PCS) and Mental Component Summary (MCS) scores. Participants completed the questionnaire independently, and scoring was conducted as per standard SF-12 scoring guidelines (15).

##### 2.5.2.3 Serum testosterone (total and free)

Fasting venous blood samples were collected between 08:00–10:00 AM to minimize diurnal variation. Serum total and free testosterone levels were quantified using an automated electro chemiluminescence immunoassay (ECLIA) on a Roche Modular Analytics E 170 analyzer (Roche Diagnostics, Mannheim, Germany).

##### 2.5.2.4 Semen analysis

Standard semen analysis was conducted at baseline and Week 8 (16). Participants provided semen samples via masturbation in a private collection room after ≥2 days of sexual abstinence. Parameters assessed included Semen volume (mL), Sperm concentration (×10^6^ /mL), Total sperm count per ejaculate (×10^6^), and sperm motility (%). All semen assessments were conducted within 30–60 min of sample collection using calibrated equipment, including an Olympus CX43 phase-contrast microscope, Makler counting chamber (Sefi-Medical Instruments, Israel), 37°C dry incubator, Eppendorf variable-volume micropipettes, and WHO-standardized glass slides and cover slips. All semen analyses were performed by a single trained laboratory technician using standardized procedures to minimize inter-observer variability.

#### 2.5.3 Safety outcomes

##### 2.5.3.1 Adverse events assessment

The number and proportion of Treatment-Emergent Adverse Events (TEAEs) and Treatment-Emergent Serious Adverse Events (TESAEs) occurring during the study period were systematically recorded to evaluate the clinical safety of the intervention. All adverse events were assessed by the investigator for severity, seriousness, onset, duration, and causality in relation to the study product. Severity was graded as mild (transient symptoms requiring no treatment), moderate (discomfort interfering with daily activities and requiring minimal intervention), or severe (significant limitation of daily activities or requiring therapeutic intervention). For each AE, the investigator also determined the likelihood of its relationship to the investigational product (unrelated, unlikely, possible, probable, or definite). All TESAEs, if any had occurred, were to be reported immediately to the Ethics Committee as per regulatory requirements. Routine physical examinations, vital signs, and laboratory parameters were monitored to identify any subclinical or emerging safety concerns.

##### 2.5.3.2 Laboratory parameters assessment

Clinical laboratory safety assessments were performed at baseline and Week 8. Following an overnight fast (8–10 h), approximately 8–10 mL of venous blood was drawn from the antecubital vein by a certified phlebotomist using sterile vacutainer tubes (EDTA for hematology and SST for biochemistry). Samples were processed within 2 h; EDTA tubes were gently mixed and analyzed immediately, while SST tubes were allowed to clot for 20–30 min, centrifuged at 3,000 rpm for 10 min at 4 °C, and serum was analyzed or stored at 2–8 °C for up to 24 h. Hemoglobin was measured using an automated hematology analyzer (e.g., Sysmex XN-1000) based on the cyanmethemoglobin method. Biochemical evaluations including creatinine (enzymatic method), BUN (urease–GLDH method), total and direct bilirubin (diazo method), alanine transaminase (ALT) and aspartate transaminase (AST) (IFCC UV-kinetic assay), alkaline phosphate (ALP) (pNPP kinetic method), total protein (biuret method), albumin (bromocresol green method), calculated globulin, and A/G ratio were performed on a fully automated chemistry platform (e.g., Roche Cobas c311/Abbott Architect). All analyses were conducted at an NABL/ISO 15189-accredited central laboratory using standardized calibrations and multi-level internal quality control procedures, and results were electronically transferred to the study database via the laboratory information management system.

### 2.6 Compliance assessment

Assessment of treatment compliance was performed through capsule count reconciliation at each study visit. Compliance was calculated as the proportion of capsules consumed relative to the number dispensed, and participants who consumed ≥80% of the scheduled doses were classified as compliant according to the study protocol.

### 2.7 Statistical methods

Sample size estimation was based on effect size data from a previously published randomized clinical trial on sexual desire in men using a polyherbal formulation (26), due to limited Ashwagandha-specific SDI-2 data at the time of study design, which is acknowledged as a methodological limitation. The study was powered to detect a 24.12-point difference in SDI-2 total score with 80% power using a one-sided alpha of 2.5%, reflecting a directional efficacy hypothesis, while all efficacy analyses were conducted using two-sided tests. To obtain 76 evaluable subjects (38 per group), 112 men were enrolled to account for attrition, with 76 participants completing the study. Statistical analyses were performed using MedCalc® Statistical Software v20.015. Efficacy outcomes (SDI-2, SSEs, IIEF, and SF-12 QoL) were analyzed in both ITT and PP populations using two-sample t-tests and repeated-measures ANOVA, with results expressed as mean ± SD and mean changes with 95% CIs; effect sizes were reported as Cohen's d. Safety outcomes were compared using the Chi-square test, normality assumptions were verified prior to parametric testing, and no adjustment for multiple comparisons was applied due to the exploratory nature of secondary endpoints.

## 3 Results

As all randomized participants completed the study without major protocol deviations, the ITT and PP populations were identical, resulting in equivalent outcomes.

### 3.1 Baseline data

**Figure 1:**
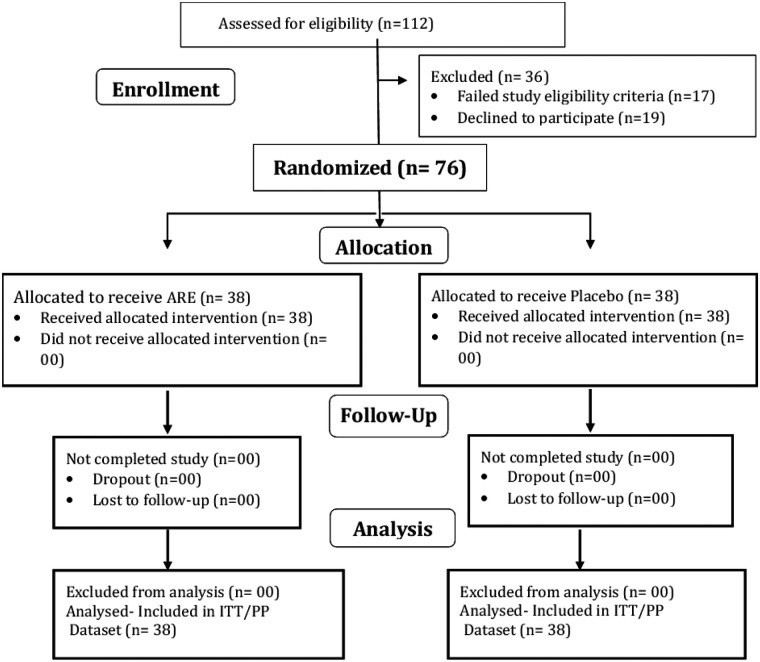
CONSORT 2010 flow diagram.

A total of 112 men were screened, of whom 76 eligible participants were enrolled and randomized into the study (CONSORT flow diagram, Figure 1). All 76 randomized participants (38 in the ARE group and 38 in the placebo group) received the study medication and were included in the ITT dataset, which served as the safety population. The PP dataset, used for efficacy analyses, also comprised all 76 participants, as each subject completed the study without major protocol deviations. Table 1 presents the demographic characteristics and baseline outcome measures for the ITT/PP population (*n* = 76). The two treatment groups were comparable at baseline with respect to demographic variables and all clinical assessment scores. None of the participants had any reported co-morbid medical conditions at study entry. Baseline sexual activity frequency was verified through participant self-report during screening interviews, while follow-up sexual activity was recorded using daily sexual activity diaries.

**Table 1:** Demography and baseline assessments.

Baseline parameters	ARE (*n* = 38)	Placebo (*n* = 38)	*p* *
	Mean (SD)	Mean (SD)	
Age (years)	39.79 (5.57)	38.74 (5.39)	0.405
BMI (kg/m^2^)	24.12 (1.10)	23.79 (1.22)	0.220
Vital parameters			
SBP (mmHg)	121.00 (3.72)	120.74 (5.75)	0.814
DBP (mmHg)	78.45 (4.51)	79.82 (6.14)	0.272
Pulse rate (per min)	79.03 (5.96)	78.50 (6.47)	0.713
Temperature (0C)	36.61 (0.61)	36.48 (0.68)	0.389
Respiratory rate (per min.)	19.26 (2.73)	19.42 (2.39)	0.789
Seminogram parameters			
Semen volume (mL)	2.37 (1.02	3.14 (4.96)	0.353
Total sperm motility (%)	23.61 (31.43)	19.95 (28.92)	0.509
Sperm concentration (million/mL)	53.18 (85.01)	52.68 (75.37)	0.943
Total sperm count (million per ejaculation)	111.67 (23.38)	115.14 (24.71)	0.851
Sexual desire inventory-2 (SDI-2)			
SDI-total score	17.21 (9.49)	17.24 (10.71)	0.265
SF-12 total score			
SF-12 total score (MCS)	39.63 (6.78)	39.49 (6.71)	0.649
SF-12 total score (PCS)	39.84 (6.84)	36.48 (6.97)	0.037
International index of erectile function (IIEF)			
IIEF-total score	13.21 (1.71)	13.26 (2.41)	0.913
Serum sex hormones			
Serum testosterone Total (ng/dl)	782.04 (316.77)	701.17 (253.36)	0.223
Serum testosterone Free (pg/ml)	15.73 (5.97)	13.73 (4.40)	0.099

* *p*-value was obtained using an independent two-sample t-test for mean differences between the two treatments (two-tailed, *α* < 0.05). BMI, body mass index; SBP, systolic blood pressure; DBP, diastolic blood pressure; ITT, intent-to-treat; PP, per protocol.

### 3.2 Primary outcomes

#### 3.2.1 Sexual desire inventory-2 (SDI-2)

**Figure 2:**
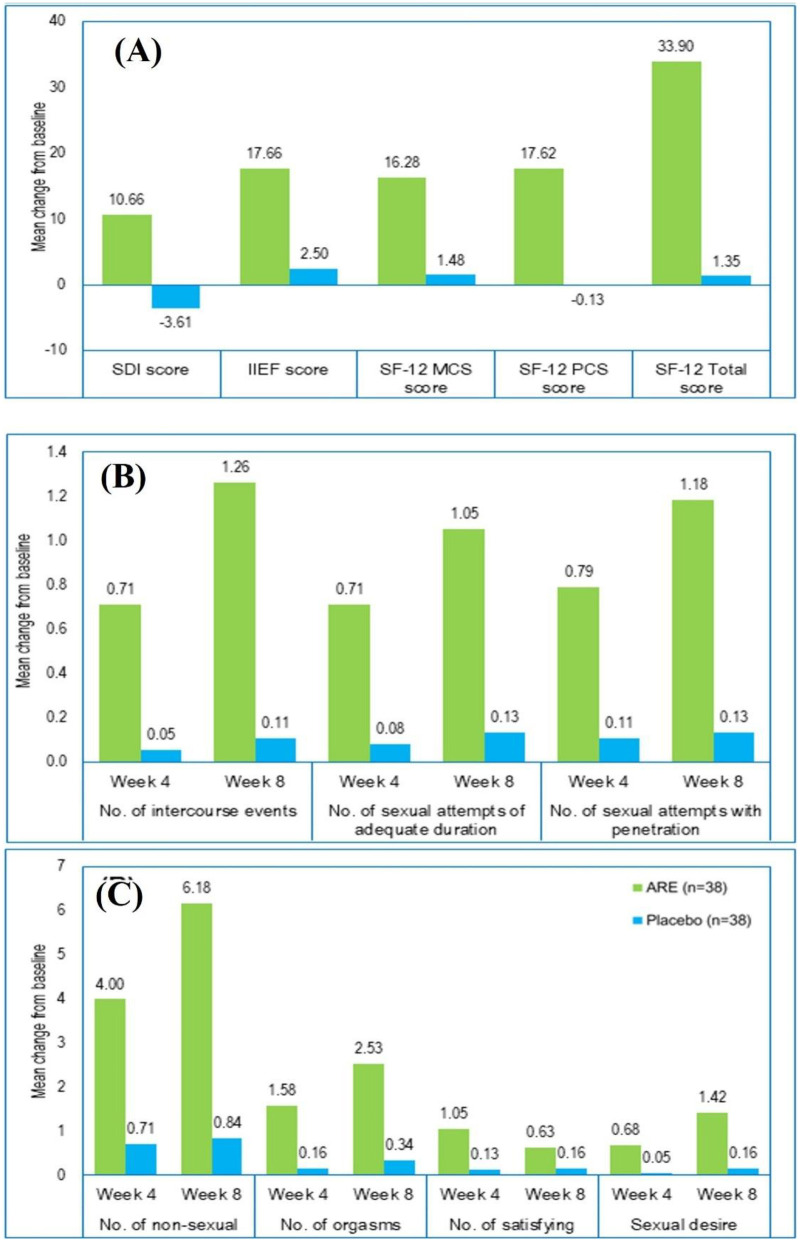
Change from baseline after week 4 and week 8. **(A)** Change from baseline in SDI-2 score, IIEF score, and SF-12 total score; (**B** and **C**) Change from baseline in SSEs.

Table 2; Figure 2A show that men in the ARE group experienced a pronounced improvement in SDI-2 scores compared with placebo. Baseline SDI-2 scores were comparable between groups (17.21 ± 9.49 vs. 17.24 ± 10.71; *p* = 0.991). By Week 8, participants receiving ARE demonstrated a substantial increase in sexual desire, with SDI-2 scores rising to 27.87 ± 12.40, whereas the placebo group showed a decline to 13.63 ± 5.86. This between-group difference was highly significant (*p* < 0.001), with a mean difference of 14.24 (95% CI: 9.81–18.67) and a large effect size (Cohen's d = 1.469). Overall, the ARE group showed a mean change of 10.66 ± 8.48 compared with −3.61 ± 11.47 in the placebo group (*p* < 0.001), confirming that ARE supplementation produced a clinically and statistically meaningful enhancement in sexual desire.SDI-2 scores increased by 61.9% over the study period.

**Table 2:** SDI-2 scores and sexual activity scores.

Parameters	ARE (*n* = 38)	Placebo (*n* = 38)	*t*-test	*Mean Difference (95% C.I.)*	Effect size
	Mean (SD)	Mean (SD)	*p*		Cohen's d
SDI-2 scale					
Baseline	17.21 (9.49)	17.24 (10.71)	0.991	−0.03 (−4.65 to 4.60)	−0.003
Week 8	27.87 (12.40)	13.63 (5.86)	<0.001	14.24 (9.81 to 18.67)	1.469
Change	10.66 (8.48)	−3.61 (11.47)	<0.001	14.26 (9.65 to 18.87)	1.414
SSE scores					
Total no. of intercourses					
Baseline	4.39 (1.33)	4.32 (1.21)	0.787	0.08 (−0.50 to 0.66)	0.062
Week 4	5.11 (1.09)	4.37 (1.34)	0.010	0.74 (0.18 to 1.30)	0.603
Week 8	5.66 (1.07)	4.42 (1.06)	<0.001	1.24 (0.75 to 1.72)	1.162
Successful duration of intercourse					
Baseline	2.47 (1.39)	2.37 (1.20)	0.724	0.11 (−0.49 to 0.70)	0.081
Week 4	3.18 (0.83)	2.45 (0.86)	<0.001	0.74 (0.35 to 1.12)	0.870
Week 8	3.53 (1.01)	2.50 (1.37)	<0.001	1.03 (0.48 to 1.58)	0.854
Success vaginal penetration					
Baseline	2.84 (1.48)	2.68 (1.25)	0.617	0.16 (−0.47 to 0.78)	0.115
Week 4	3.63 (1.38)	2.79 (1.12)	0.005	0.84 (0.27 to 1.42)	0.669
Week 8	4.03 (1.42)	2.82 (1.41)	<0.001	1.21 (0.56 to 1.86)	0.854
Total non-intercourse interactions					
Baseline	11.16 (8.29)	11.21 (8.82)	0.979	−0.05 (−3.96 to 3.86)	−0.006
Week 4	15.16 (7.31)	11.92 (9.65)	0.104	3.24 (−0.68 to 7.15)	0.378
Week 8	17.34 (5.77)	12.05 (9.38)	0.004	5.29 (1.73 to 8.85)	0.679
Total No. of orgasms					
Baseline	6.68 (4.93)	6.61 (4.94)	0.945	0.08 (−2.18 to 2.33)	0.016
Week 4	8.26 (3.86)	6.76 (5.05)	0.150	1.50 (−0.55 to 3.55)	0.334
Week 8	9.21 (3.09)	6.95 (3.88)	0.006	2.26 (0.66 to 3.87)	0.645
Total no. of SSE's					
Baseline	1.92 (1.36)	1.82 (1.14)	0.716	0.11 (−0.47 to 0.68)	0.084
Week 4	2.97 (0.85)	1.95 (1.11)	<0.001	1.03 (0.57 to 1.48)	1.034
Week 8	2.55 (0.95)	1.97 (0.97)	0.011	0.58 (0.14 to 1.02)	0.602
Sexual desire					
Baseline	0.89 (0.61)	0.87 (0.62)	0.852	0.03 (−0.25 to 0.31)	0.043
Week 4	1.58 (0.76)	0.92 (0.49)	<0.001	0.66 (0.37 to 0.95)	1.033
Week 8	2.32 (0.57)	1.03 (0.54)	<0.001	1.29 (1.03 to 1.55)	2.304

ARE, ashwagandha; BMI, body mass index; C.I., confidence interval; SEM, standard error for mean; SD, standard deviation; SSE, satisfying sexual events; SDI, sexual desire inventory.

#### 3.2.2 Satisfying sexual events (SSEs)

SSE scores did not differ between groups at baseline (Table 2; Figures 2B,C). By Week 4, ARE produced a significant improvement in the total number of intercourses compared with placebo (*p* = 0.010), which further increased at Week 8 [*p* < 0.001; mean difference (MD) = 1.24; 95% CI: 0.75–1.72]. The successful duration of intercourse was comparable at baseline, but significantly favoured ARE at Week 4 (*p* < 0.001) and Week 8 (*p* < 0.001; MD = 1.03; 95% CI: 0.48–1.58). ARE showed significantly greater improvement in successful vaginal penetration at Week 4 (*p* = 0.005) and at Week 8 (*p* < 0.001; MD = 1.21; 95% CI: 0.56–1.86). For non-intercourse intimate interactions, a non-significant trend was observed at Week 4 (*p* = 0.104). By Week 8, ARE demonstrated a significant increase over placebo (*p* = 0.004; MD = 5.298; 95% CI: 1.73–8.85). The changes in Week 4 were not statistically significant for the total number of orgasms (*p* = 0.150). By Week 8, ARE produced a significant improvement for the total number of orgasms (*p* = 0.006; MD = 2.26; 95% CI: 0.66–3.87). ARE showed significant increases in the total number of SSEs at Week 4 (*p* < 0.001) and Week 8 (*p* = 0.011; MD = 0.58; 95% CI: 0.14–1.02). Significant improvements were observed in sexual desire in the ARE group at Week 4 (*p* < 0.001) and more prominently at Week 8 (*p* < 0.001; MD: 1.29; 95% CI: 1.03–1.55). From baseline to Week 8, the ARE group demonstrated substantial within-group improvements across all domains. SSE scores improved by 28.9%, successful duration of intercourse by 42.9%, and successful vaginal penetration by 41.9%. Non-intercourse intimate interactions increased by 55.4%, while the total number of orgasms and SSEs increased by 37.9% and 32.8%, respectively. The greatest improvement was observed in sexual desire over the 8-week period.

### 3.3 Secondary outcomes

#### 3.3.1 International Index of erectile function (IIEF)

IIEF-total scores were comparable between groups at baseline (*p* = 0.913). By Week 8, the ARE group demonstrated a significant improvement compared with placebo (*p* < 0.001), with an MD of 15.11 (95% CI: 11.30–18.91). The change-from-baseline analysis similarly showed a markedly greater increase in the ARE group (*p* < 0.001). The magnitude of improvement was substantial, as evidenced by very large effect sizes (Cohen's d > 1.7).

#### 3.3.2 Quality of life (QoL)

Quality of life was assessed using the SF-12 survey, which includes MCS, PCS, and an overall Total Score (Table 3). MCS scores were comparable between groups at baseline (*p* = 0.929). By Week 8, the ARE group demonstrated a significant improvement over placebo (*p* < 0.001), with an MD of 14.94 (95% CI: 12.49–17.38) and a very large effect size (d = 2.797). Change-from-baseline analysis similarly favored ARE (*p* < 0.001). PCS scores did not differ at baseline (*p* = 0.873). At Week 8, ARE supplementation led to an improvement compared with placebo (*p* < 0.001), with an MD of 17.96 (95% CI: 15.55–20.38). The change-from-baseline analysis revealed even greater differences (*p* < 0.001; MD: 17.75, 95% CI: 16.09–19.41; d = 4.89). Total QoL scores were comparable at baseline (*p* = 0.879). By Week 8, the ARE group showed a highly significant overall improvement (*p* < 0.001), with an MD of 32.90 (95% CI: 29.00–36.80). Change-from-baseline results also strongly favoured ARE (*p* < 0.001; d = 3.99). The SF-12 assessment showed meaningful improvements across all domains, with a 41.1% increase in the MCS score, a 47.7% increase in the PCS score, and a 44.3% improvement in the overall SF-12 Total Score.

**Table 3:** IIEF and SF−12 scores.

Parameters	ARE (*n* = 38)	Placebo (*n* = 38)	*t*-test	*Mean Difference (95% C.I.)*	Effect size
	*Mean (SD)*	*Mean (SD)*	*p*		*Cohen's d*
IIEF-total score					
Baseline	13.21 (1.71)	13.26 (2.41)	0.913	−0.05 (−1.01 to 0.90)	−0.025
Week 8	30.87 (10.55)	15.76 (5.22)	<0.001	15.11 (11.30 to 18.91)	1.815
Change from baseline	17.66 (11.07)	2.50 (5.29)	<0.001	15.16 (11.19 to 19.12)	1.747
SF-12 MCS score					
Baseline	39.63 (6.78)	39.49 (6.71)	0.929	0.14 (−2.95 to 3.22)	0.020
Week 8	55.91 (4.92)	40.98 (5.73)	<0.001	14.94 (12.49 to 17.38)	2.797
Change from baseline	16.28 (8.96)	1.48 (5.10)	<0.001	14.80 (11.47 to 18.13)	2.031
SF-12 PCS score					
Baseline	36.95 (4.29)	36.74 (6.86)	0.873	0.21 (−2.41 to 2.83)	0.037
Week 8	54.57 (2.87)	36.60 (6.89)	<0.001	17.96 (15.55 to 20.38)	3.402
Change from baseline	17.62 (4.82)	−0.13 (1.76)	<0.001	17.75 (16.09 to 19.41)	4.892
SF-12 Total score					
Baseline	76.58 (7.97)	76.23 (11.55)	0.879	0.35 (−4.19 to 4.88)	0.035
Week 8	110.48 (6.27)	77.58 (10.30)	<0.001	32.90 (29.00 to 36.80)	3.859
Change from baseline	33.90 (10.06)	1.35 (5.60)	<0.001	32.55 (28.83 to 36.27)	3.998

ARE, ashwagandha, C.I., confidence interval, SEM, standard error for mean; SD, standard deviation; IIEF, international index of erectile function; MCS, mental component score; PCS, physical component score.

#### 3.3.3 Serum testosterone (total and free)

Total testosterone levels were comparable at baseline (*p* = 0.947). By Week 8, the increase in the ARE group did not differ significantly from placebo (*p* = 0.223; MD = 80.87, 95% CI: −50.24 to 211.98; d = 0.28). Free testosterone concentrations were similar at baseline (*p* = 0.957). Although higher values were observed in the ARE group at Week 8, the between-group difference did not reach statistical significance (*p* = 0.099; MD = 2.01, 95% CI: −0.39 to 4.40; d = 0.84) (Table 4).

**Table 4:** Serum testosterone and seminogram parameters.

Parameters	ARE (*n* = 38)	Placebo (*n* = 38)	*t*-test	*Mean Difference (95% C.I.)*	Effect size
	*Mean (SD)*	*Mean (SD)*	*p*		*Cohen's d*
Serum Testosterone Total (ng/dl)					
Baseline	697.17 (337.43)	692.59 (258.50)	0.947	4.58 (−132.82 to 141.97)	0.015
Week 8	782.04 (316.77)	701.17 (253.36)	0.223	80.87 (−50.24 to 211.98)	0.282
Serum testosterone free (pg/ml)					
Baseline	14.27 (6.21)	14.21 (4.24)	0.957	0.07 (−2.36 to 2.50)	0.462
Week 8	15.73 (5.97)	13.73 (4.40)	0.099	2.01 (−0.39 to 4.40)	0.836
Semen volume (mL)					
Baseline	2.47 (0.84)	2.37 (0.64)	0.572	0.10 (−0.24 to 0.44)	0.130
Week 8	3.37 (0.67)	2.29 (0.62)	<0.001	1.08 (0.79 to 1.38)	1.680
Total sperm motility (%)					
Baseline	18.18 (16.32)	17.89 (16.92)	0.940	0.29 (−7.31 to 7.89)	0.467
Week 8	34.03 (25.48)	17.95 (16.52)	0.002	16.08 (6.26 to 25.90)	1.212
Sperm concentration (million/mL)					
Baseline	53.18 (31.43)	52.68 (28.92)	0.943	0.49 (−13.31 to 14.30)	0.016
Week 8	70.67 (31.11)	53.45 (28.08)	0.013	17.21 (3.67 to 30.76)	0.581
Total sperm count (million per ejaculation)					
Baseline	115.58 (83.20)	115.14 (75.37)	0.981	0.43 (−35.85 to 36.72)	0.455
Week 8	159.57 (82.00)	115.37 (72.09)	0.015	44.19 (8.90 to 79.48)	1.030

ARE, ashwagandha, C.I., confidence interval; SEM, standard error for mean; SD, standard deviation; ng/dl, nanogram per decilitre; pg./dl, picogram per liter.

#### 3.3.4 Semen analysis

Baseline semen volume was comparable between groups (*p* = 0.572) (Table 4). By Week 8, ARE supplementation produced a significant increase in semen volume relative to placebo (*p* < 0.001), with an MD of 1.08 (95% CI: 0.79–1.38). Groups did not differ at baseline (*p* = 0.940) for total sperm motility. At Week 8, total sperm motility was significantly higher in the ARE group (*p* = 0.002), with an MD of 16.08 (95% CI: 6.26–25.90; d = 1.21). By Week 8, ARE resulted in a significant improvement in sperm concentration compared with placebo (*p* = 0.013), with an MD of 17.21 (95% CI: 3.67–30.76; d = 0.581). By Week 8, ARE supplementation produced a significant increase in total sperm count per ejaculation (*p* = 0.015), with an MD of 44.19 (95% CI: 8.90–79.48) and a large effect size (d = 1.03).

Participants receiving ARE demonstrated marked improvements across all key semen parameters over the 8-week period. Semen volume increased by 36.4%, while sperm concentration rose by 32.9%. A 38.1% increase in total sperm count per ejaculation, and total sperm motility improved by 87.25%.

### 3.4 Safety outcomes

#### 3.4.1 Adverse events assessments

No adverse events or serious adverse events were reported during study by the patients.

#### 3.4.2 Laboratory parameters assessment

Biochemical assessments demonstrated that ARE was well tolerated over the 8-week intervention period, with no clinically meaningful safety concerns. Hemoglobin levels increased significantly in the ARE group compared with placebo (*p* = 0.015). Renal markers showed a significant reduction in serum creatinine with ARE (*p* = 0.023), whereas the placebo group showed no comparable improvement. Blood urea nitrogen (BUN) exhibited a significant decline in the ARE group (*p* < 0.001), while it increased in the placebo group, further suggesting supportive renal effects.

Liver function parameters, including total bilirubin, ALT, AST, and ALP, remained within normal physiological limits in both groups throughout the study. Notably, ARE resulted in significant reductions from baseline in total bilirubin (*p* < 0.001), ALT (*p* < 0.001), AST (*p* < 0.001), and ALP (*p* < 0.001), whereas the placebo group demonstrated slight increases. Serum protein fractions showed minimal changes across groups; however, globulin levels decreased significantly in the ARE group (*p* = 0.001), contributing to stable A/G ratios over the study period (Table 5).

**Table 5:** Laboratory parameters.

Parameters		Baseline			Week 8			Change from baseline		
	Treatment	Mean	SD	*p*	Mean	SD	*p*	Mean	SD	*p*
Hb (gm/dL)	ARE (*n* = 34)	13.48	0.54	0.200	14.22	0.44	0.013	0.75	0.31	0.015
	Placebo (*n* = 35)	13.31	0.52		13.89	0.61		0.56	0.33	
Creatinine (mg/dL)	ARE (*n* = 34)	0.76	0.14	0.941	0.59	0.24	0.095	−0.17	0.19	0.023
	Placebo (*n* = 35)	0.75	0.14		0.69	0.24		−0.07	0.17	
BUN (mg/dL)	ARE (*n* = 34)	18.32	3.30	0.809	18.22	3.30	0.099	−0.10	0.00	<0.001
	Placebo (*n* = 35)	18.12	3.66		19.57	3.41		1.45	1.62	
Bilirubin total (mg/dL)	ARE (*n* = 34)	0.58	0.30	0.511	0.48	0.30	0.512	−0.10	0.00	<0.001
	Placebo (*n* = 35)	0.53	0.31		0.53	0.31		0.00	0.10	
Bilirubin Direct (mg/dL)	ARE (*n* = 34)	0.29	0.13	0.933	0.27	0.16	0.299	−0.02	0.09	0.097
	Placebo (*n* = 35)	0.28	0.14		0.23	0.15		−0.05	0.09	
Protein (gm/dL)	ARE (*n* = 34)	6.72	6.08	0.589	6.45	6.06	0.641	−0.27	0.12	0.052
	Placebo (*n* = 35)	5.85	7.10		5.71	7.03		−0.14	0.35	
Albumin (gm/dL)	ARE (*n* = 34)	4.18	3.04	0.598	4.01	3.02	0.635	−0.17	0.13	0.330
	Placebo (*n* = 35)	3.76	3.56		3.63	3.53		−0.13	0.23	
Globulin (gm/dL)	ARE (*n* = 34)	2.54	3.03	0.579	2.45	3.03	0.647	−0.10	0.01	0.001
	Placebo (*n* = 35)	2.10	3.54		2.08	3.50		−0.02	0.13	
A-G Ratio	ARE (*n* = 34)	1.45	0.25	0.879	1.45	0.26	0.960	−0.01	0.04	0.210
	Placebo (*n* = 35)	1.46	0.28		1.44	0.28		−0.02	0.05	
ALT (IU/L)	ARE (*n* = 34)	29.12	3.30	0.809	29.02	3.30	0.099	−0.10	0.00	<0.001
	Placebo (*n* = 35)	28.92	3.66		30.37	3.41		1.45	1.62	
AST (IU/L)	ARE (*n* = 34)	29.32	3.30	0.809	29.22	3.30	0.099	−0.10	0.00	<0.001
	Placebo (*n* = 35)	29.12	3.66		30.57	3.41		1.45	1.62	
ALP (U/L)	ARE (*n* = 34)	87.36	9.90	0.809	87.26	9.90	0.702	−0.10	0.00	<0.001
	Placebo (*n* = 35)	86.75	10.97		88.21	10.48		1.45	1.62	

p*, value was obtained using an unpaired *t*-test; SD., standard deviation; ARE, ashwagandha root extract; SOC, standard of care; Hb, hemoglobin; ALT, alanine transaminase; AST, aspartate transaminase; ALP, alkaline phosphatase; BUN, blood urea nitrogen; A-G Ratio, albumin-to-globulin; Bilirubin Total, total protein in grams per deciliter.

### 3.5 Compliance assessment

Treatment compliance was high in both groups throughout the intervention period. Based on capsule count reconciliation, participants in the ARE and placebo groups consumed the study capsules consistently, with the majority achieving ≥80% adherence as defined in the protocol.

## 4 Discussion

The present randomized, double-blind, placebo-controlled clinical trial investigated the effects of an 8-week supplementation with ARE on multiple domains of male sexual health, reproductive parameters, hormonal balance, and overall quality of life. While a complete spermatogenic cycle spans approximately 64–72 days, the 8-week intervention period was selected to detect early and functionally relevant changes in semen parameters, as reported in previous Ashwagandha studies. The study demonstrated robust and consistent improvements across nearly all evaluated endpoints. Participants receiving ARE showed statistically significant gains in erectile function (IIEF), sexual desire (SDI-2), sexual activity frequency, orgasmic function, and partner-related sexual outcomes. These functional improvements were accompanied by measurable enhancements in physiological markers, including serum testosterone (total and free), semen volume, sperm concentration, total sperm count, and sperm motility, alongside significant improvements in SF-12 physical and mental component scores. Together, these results indicate that ARE exerts a multi-dimensional effect on male sexual and reproductive health.

The observed improvements can be mechanistically explained through the adaptogenic, endocrine-modulating, and rejuvenating properties of Ashwagandha root (17). Chronic stress is one of the major contributors to sexual dysfunction, primarily due to elevated cortisol levels that impair testosterone production, reduce sexual desire, and cause fatigue-associated inhibitory effects on sexual activity (18). Several clinical studies have confirmed Ashwagandha's ability to significantly reduce serum cortisol and improve stress resilience and sleep quality (17, 19, 20). These psychophysiological improvements likely contributed to the enhanced sexual desire, increased intercourse frequency, and improved orgasmic ability seen in the current study.

Additionally, Ashwagandha is reported to enhance energy, stamina, and endurance, supporting improved physical readiness for sexual activity. Increased testosterone levels in prior studies, aligns directly with the significant increases in testosterone observed in our participants (9, 11). Testosterone plays a central role in libido, erectile rigidity, sperm production, and sexual satisfaction, and thus its elevation is biologically consistent with the improvements across sexually relevant outcomes (16, 23). Although numerical increases in total and free testosterone were observed, between-group differences were not statistically significant; therefore, hormonal findings should be interpreted as exploratory and supportive rather than confirmatory.

ARE supplementation resulted in substantial improvements in IIEF total scores and SDI-2 scores, reflecting enhanced erectile function, desire, libido, and sexual satisfaction. Increases in total number of intercourses, successful duration of intercourse, successful vaginal penetration, and orgasm frequency further demonstrate functional and behavioral improvements, not just subjective perceptions.

Participants receiving ARE exhibited notable increases in total and free testosterone, supporting Ashwagandha's androgen-enhancing properties. These hormonal improvements provide a biological foundation for the sexual function benefits observed.

ARE produced marked improvements in semen volume (+36.4%), sperm concentration (+32.9%), total sperm count (+38.1%), and total sperm motility (+87.25%). Such changes indicate enhanced spermatogenesis, improved seminal quality, and potentially greater fertility potential.

Significant increases in SF-12 mental and physical component scores suggest that ARE exerts broad benefits extending beyond sexual health, likely driven by improved stress adaptation, reduced fatigue, better sleep, and enhanced psychological well-being.

The findings of our study are consistent with previous research demonstrating Ashwagandha's beneficial effects on stress, sexual function, and hormonal balance. Salve et al. reported significant reductions in PSS scores and improvements in sleep quality with Ashwagandha supplementation, supporting our observation of improved QOL and sexual desireboth of which are highly stress-linked (17).

Chauhan N. et al. highlighted Ashwagandha's aphrodisiac, adaptogenic, and tonic properties in their review of plants improving sexual performance, aligning with the multi-domain improvements observed here (24). Similarly, Chauhan S. et al. demonstrated significant improvements in DISF-M scores and serum testosterone levels with ARE supplementation, closely mirroring our findings (21). Contrary to the study by Mamidi P. et al., which reported no significant improvements in psychogenic ED with Ashwagandha tablets, the present study showed robust improvements in IIEF erectile function scores (*p* < 0.001) (25). This discrepancy may relate to differences in extract composition, dosage, participant characteristics, or severity of baseline dysfunction. Additionally, Dongre et al. demonstrated improved sexual function in healthy females with high-concentration Ashwagandha root extract, further supporting the cross-gender relevance of Ashwagandha's adaptogenic sexual health benefits (22).

This study possesses several noteworthy strengths. It employed a randomized, double-blind, placebo-controlled design with well-defined eligibility criteria, minimizing selection and performance bias. The use of standardized Ashwagandha root extract and validated outcome measures including semen analysis, serum testosterone assays, and the SF-12 quality-of-life survey enhances both methodological rigor and clinical relevance. High treatment compliance further supports the reliability of the findings. Additionally, the study evaluated multiple mechanistic and clinical endpoints, providing a comprehensive understanding of Ashwagandha's effects on male reproductive health.

Several limitations should be acknowledged. The study duration 8 weeks while sufficient may not adequately capture long-term reproductive or endocrine effects, and the modest sample size limits generalizability to broader or more diverse populations. Lifestyle factors such as diet and physical activity were not formally quantified, introducing potential residual confounding. Mechanistic insights were limited, as advanced biomarkers, including oxidative stress markers, inflammatory cytokines, and reproductive hormones other than testosterone were not assessed. The requirement for partner availability may have biased recruitment toward individuals in stable relationships, further affecting generalizability. Additionally, baseline sexual activity relied on recall-based reporting, whereas follow-up data used daily diaries, which may affect comparability. Despite these constraints, the findings provide supportive evidence for the beneficial effects of Ashwagandha root extract on male reproductive and hormonal outcomes.

## 5 Conclusion

The study observed noteworthy enhancements in the sexual activity scores and semen parameters with 8-week oral administration of Ashwagandha Root Extract in healthy men visiting infertility center. Furthermore, a substantial improvement has been observed in the scores of both the SDI-2 and the IIEF, and satisfaction with intercourse. It is noted that Ashwagandha was well tolerated with no adverse events reported by the participants, and no changes in hepatic and renal parameters in blood.

## Data Availability

The raw data supporting the conclusions of this article will be made available by the authors, without undue reservation.
